# Pulmonary Embolism following Cessation of Infliximab for Treatment of Miliary Tuberculosis

**DOI:** 10.1155/2014/479025

**Published:** 2014-10-29

**Authors:** Brian Lee, Farid Moosavy

**Affiliations:** ^1^Thomas Jefferson University, 1025 Walnut Street, Philadelphia, PA 19107, USA; ^2^Attending, Pulmonary and Critical Care Section, Christiana Care Health System, 4745 Ogletown-Stanton Road, Map 1, Suite 220, Newark, DE 19713, USA

## Abstract

We report a case of a 41-year-old male who presented with tachycardia and swelling of his left arm six weeks after he started antituberculosis treatment and stopped his rheumatoid arthritis infliximab treatment. He was diagnosed with pulmonary embolism by chest CT and initially treated with warfarin, which interacted with his antituberculosis treatment. This presentation of deep vein thrombosis and pulmonary embolism as part of immune reconstitution inflammatory syndrome has not been previously reported for infliximab treated patients.

## 1. Introduction

Biologic agents are increasingly used as disease-modifying antirheumatic drugs (DMARDS) because of their ability to slow down progression of the disease. However, their use can lead to increased susceptibility to tuberculosis. Tuberculosis treatment, in turn, typically involves elimination of immunosuppression by these biologic agents. Rapid changes in the immune system can lead to a heightened and generalized inflammatory response, usually referred to as immune reconstitution inflammatory syndrome.

## 2. Case Presentation

A 41-year-old male, a recent immigrant, presented to the emergency room with swelling and pain in his neck and left arm. He had a history of rheumatoid arthritis and started treatment with infliximab, methotrexate, and high dose prednisone one year prior to admission. Six weeks prior to admission, he developed night sweats and weight loss, which was diagnosed as miliary tuberculosis by CT imaging and positive sputum culture. His tuberculosis was treated with rifampin, pyrazinamide, isoniazid, and ethambutol. His anti-TNF and steroid therapy were discontinued and replaced with hydroxychloroquine and sulfasalazine. The day before his admission, the patient noticed swelling and pain in his left neck and mild dyspnea. The swelling progressed down his entire arm over the course of the day. The patient was a lifelong nonsmoker and had no prior history of deep vein thrombosis (DVT).

On physical examination, he had a BMI of 24 and was afebrile but tachycardic to 130. He had cervical and supraclavicular lymphadenopathy. His entire left arm was erythematous, swollen, and tender to palpation. Range of motion of that extremity was reduced secondary to pain.

**Figure 1:**
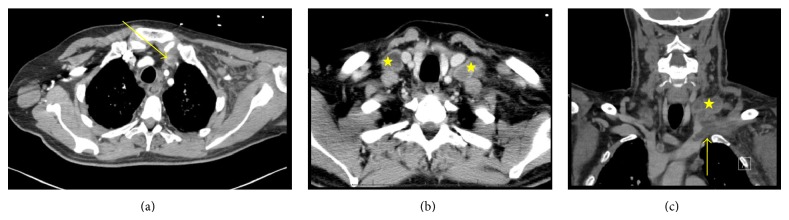
CT angiography showing (a) thrombus (arrow) in the left subclavian vein extending into the proximal innominate. Bilateral necrotic supraclavicular lymphadenopathy (star) does not show compression of the subclavian vein in the (b) axial and (c) coronal views.

**Figure 2:**
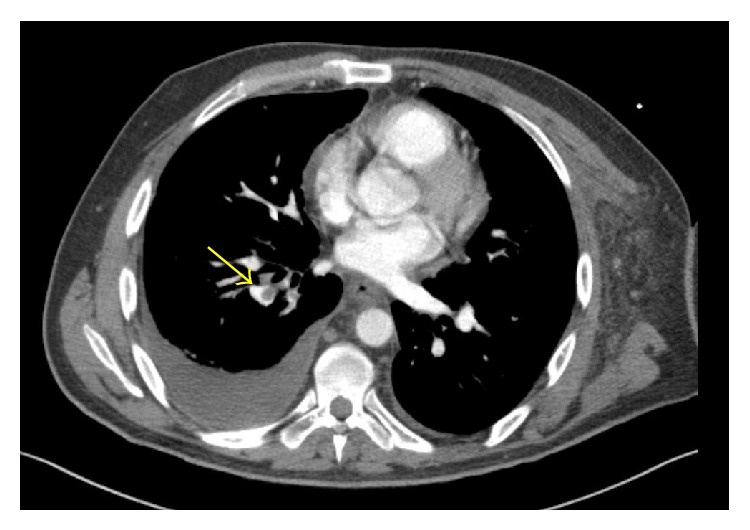
CT angiography demonstrating lobar and segmental pulmonary emboli (arrow) in the right lower lobe.

CT of neck revealed a thrombus within the left axillary and left subclavian veins extending to the proximal left brachiocephalic vein, in addition to enlarged right and left supraclavicular lymph nodes, without obvious vascular compression (Figure 1). CT angiography of chest showed pulmonary emboli involving the right lower lobe in addition to previously noted nodular opacities of miliary tuberculosis (Figure 2).

His hemogram was significant for normocytic anemia and neutrophilia. Electrolytes, renal and liver function tests, hepatitis panels, and blood cultures were normal and his acid-fast smear was now negative. His coagulation profile was normal except for an elevated D-dimer of greater than 1000 ng/mL. An extensive work-up for thrombophilia was unremarkable with normal lupus anticoagulant, silicone-clotting test, Factor V Leiden, prothrombin 20210, Cardiolipin IgG, IgM 5, and beta-2 glycoprotein.

The patient was treated with enoxaparin and his swelling and dyspnea subsided. A warfarin regimen was attempted at 5 mg, but his INR remained 1.2. The patient was subsequently discharged being on rivaroxaban, prednisone 20 mg, and trimethoprim-sulfonamide prophylaxis, in addition to his antituberculosis regimen.

## 3. Discussion

It is well known that disease-modifying antirheumatics, such as infliximab, significantly increase the risk of tuberculosis [1]. Patients receiving interferon and adalimumab are more at risk compared to those receiving etanercept [2]. Recent reports indicate cumulative incidence rates as high as 499/100,000 in rheumatoid arthritis and 287/100,000 in inflammatory bowel disease [3].

Thus, both active and latent tuberculosis are contraindications to the use of anti-TNF-alpha drugs, and candidates for infliximab therapy are screened with a tuberculin skin test, which, for this group, is positive if it is greater than 6 mm [4]. However, these patients are often on anti-inflammatory medication such as steroids and thus are more likely to have negative tuberculin skin tests [5].

Immune reconstitution inflammatory syndrome (IRIS) has been well described in HIV-positive patients upon initiation of antiretroviral therapy [6]. IRIS has also been reported in patients on infliximab who develop tuberculosis [7, 8]. There is a five-to-sixteen-week period between cessation of infliximab treatment and onset of symptoms, such as progression of lymph node swelling, infiltrates, or pleural effusion [9].

To our knowledge, however, the development of pulmonary thromboembolic disease has never been described in the setting of IRIS specifically occurring as a consequence of discontinuation of anti-TNF therapy. We speculate that this complication was the result of concurrent biomechanical and biochemical factors as delineated below.

Virchow's triad has been used to explain the association of lower extremity DVT with TB in multiple reports [10]. In our case, lymph node enlargement could have mechanically contributed to this unusual case of upper extremity DVT, presumably with impeding the flow in the adjacent veins at least in certain positions. Cervical lymphadenopathy is the most common lymphadenopathy in TB [11], but any lymphatic chain, including the supraclavicular lymph nodes, can easily be involved in the setting of generalized inflammatory resurgence as part of immune reconstitution as in this case.

Both RA and TB could have presumably contributed to a hypercoagulable state in this patient. Early in tuberculosis, procoagulants such as fibrin degradation products (FDP) and tissue plasminogen activator (t-PA) are increased [12]. Although their levels normalize over the course of 12 weeks of treatment, they can still be susceptible to DVT [12]. In a review of autopsies in 1948, Zahn and Peirce found that 1.5% of tuberculosis subjects had DVT and less than 0.1% had pulmonary embolism as the cause of death [13]. In a 2013 review of 30 subjects who had DVT after receiving antituberculosis therapy, Kouismi et al. documented 5 cases of pulmonary tuberculosis that resulted in pulmonary embolism [10]. While pulmonary embolism is a rare complication of tuberculosis, rheumatoid arthritis is also an autoinflammatory disorder associated with endothelial dysfunction through the rise in immune mediators and cytokines and can have similar thromboembolic complications [14].

Overall, in our patient, the reduction in RA treatment and initiation of antituberculous treatment could have conceivably created an immune reconstitution inflammatory syndrome leading to a procoagulant milieu. To our knowledge, this is the first report of a pulmonary thromboembolic manifestation of immune reconstitution inflammatory syndrome as a result of withdrawal of infliximab treatment following miliary tuberculosis.
